# Induction of resistance to neurotrophic tropomyosin‐receptor kinase inhibitors by HMGCS2 via a mevalonate pathway

**DOI:** 10.1002/cam4.7393

**Published:** 2024-06-24

**Authors:** Yasuhiro Kato, Masaru Matsumoto, Natsuki Takano, Mariko Hirao, Kuniko Matsuda, Takehiro Tozuka, Naomi Onda, Shinji Nakamichi, Susumu Takeuchi, Akihiko Miyanaga, Rintaro Noro, Akihiko Gemma, Masahiro Seike

**Affiliations:** ^1^ Department of Pulmonary Medicine and Oncology, Graduate School of Medicine Nippon Medical School Tokyo Japan

**Keywords:** HMGCS2, mevalonate pathway, NTRK‐TKI, resistance mechanism, statin

## Abstract

**Introduction:**

A neurotrophic tropomyosin receptor kinase (NTRK)‐tyrosine kinase inhibitor (TKI) has shown dramatic efficacy against malignant tumors harboring an *NTRK* fusion gene. However, almost all tumors eventually acquire resistance to NTRK‐TKIs.

**Method:**

To investigate the mechanism of resistance to NTRK‐TKIs, we established cells resistant to three types of NTRK‐TKIs (larotrectinib, entrectinib, and selitrectinib) using KM12 colon cancer cells with a *TPM3‐NTRK1* rearrangement.

**Result:**

Overexpression of 3‐hydroxy‐3‐methylglutaryl‐CoA synthase 2 (HMGCS2) was observed in three resistant cells (KM12‐LR, KM12‐ER, and KM12‐SR) by microarray analysis. Lower expression of sterol regulatory element‐binding protein 2 (SREBP2) and peroxisome proliferator activated receptor α (PPARα) was found in two cells (KM12‐ER and KM12‐SR) in which HMGCS2 was overexpressed compared to the parental KM12 and KM12‐LR cells. In resistant cells, knockdown of HMGCS2 using small interfering RNA improved the sensitivity to NTRK‐TKI. Further treatment with mevalonolactone after HMGCS2 knockdown reintroduced the NTRK‐TKI resistance. In addition, simvastatin and silibinin had a synergistic effect with NTRK‐TKIs in resistant cells, and delayed tolerance was observed after sustained exposure to clinical concentrations of NTRK‐TKI and simvastatin in KM12 cells. In xenograft mouse models, combination treatment with entrectinib and simvastatin reduced resistant tumor growth compared with entrectinib alone.

**Conclusion:**

These results suggest that HMGCS2 overexpression induces resistance to NTRK‐TKIs via the mevalonate pathway in colon cancer cells. Statin inhibition of the mevalonate pathway may be useful for overcoming this mechanistic resistance.

## Graphical abstract

We confirmed HMGCS2 induces resistance to NTRK‐TKIs via mevalonate pathway in vitro and vivo. Statins may overcome the resistance mechanism via the mevalonate pathway by HMGCS2.

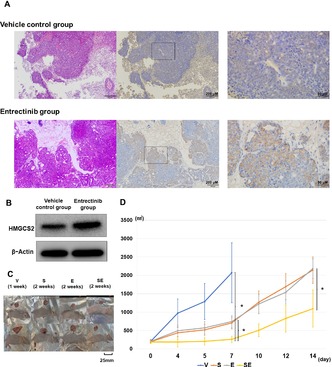

## 1 INTRODUCTION

*Neurotrophic tropomyosin receptor kinase* (*NTRK*) gene fusions are considered to be targetable oncogenic driver gene rearrangements and the most common mechanisms of oncogenic tropomyosin receptor kinase (TRK) activation.^1^, ^2^, ^3^ Three types of tropomyosin receptor kinases (TRK‐A, TRK‐B, and TRK‐C) are encoded by neurotrophic tyrosine receptor kinases *NTRK*1, *NTRK*2, and *NTRK*3, respectively.^1^, ^4^ TRK activation due to such fusions promotes the development of cancer by promoting cancer cell survival or proliferation through the activation of phosphoinositide 3‐kinase–AKT, mitogen‐activated protein kinase, and phospholipase C pathways.^3^, ^5^ Entrectinib and larotrectinib are first‐generation tyrosine kinase inhibitors (TKIs) that target *NTRK* fusion gene–positive tumors.^6^, ^7^ Previous prospective trials of tumors with *NTRK* fusions confirmed the efficacy of such agents.^8^, ^9^, ^10^, ^11^, ^12^ Molecular targeted therapies are currently considered the standard treatment for *NTRK* fusion gene–positive tumors. In addition, current clinical trials (NCT03215511) for second‐generation agents that demonstrate preclinical efficacy against even more genotype variants than first‐generation agents are underway.^13^, ^14^

However, almost all *NTRK* fusion gene–positive tumors eventually acquire resistance to molecular‐targeted therapies. Secondary mutations in the kinase domain were reported as “on‐target mechanisms” and secondary acquisitions of *MET* amplification, *KRAS* mutation, *BRAF V600E* mutation, insulin growth factor receptor type 1 bypass pathway‐mediated resistance were reported as “off‐target resistance mechanisms.”^13^, ^15^, ^16^, ^17^, ^18^ However, the elucidation of the mechanisms of resistance, especially to second‐generation drugs, remains incomplete.

In this study, we established three resistant tumor cells from a KM12 with a *TPM3‐NTRK1* rearrangement in vitro and *vivo* and analyzed the mechanisms of drug resistance.

## 2 MATERIALS AND METHODS

### 2.1 Cell culture

KM12 was kindly provided from Dr. Ryohei Katayama and his laboratory as Cancer Chemotherapy Center, Experimental Chemotherapy. Parental KM12 and resistant cells were cultured in RPMI‐1640 (Fujifilm, Osaka, Japan) containing 10% fetal bovine serum (FBS; Biowest, Nuaille, France) and 1% penicillin and streptomycin (Fujifilm) at 37°C in a 5% CO_2_ incubator. Each resistant cell line was incubated with 2 μM of the corresponding agent. All the cells were routinely screened for the absence of mycoplasma.

### 2.2 Drugs and cell viability assay

Larotrectinib, entrectinib, simvastatin, and silibinin were purchased from Selleck Chemicals (Houston, TX). Selitrectinib was purchased from MedChemExpress (Monmouth Junction, NJ). Mevalonolactone was purchased from TCI Chemicals (Tokyo, Japan), and to evaluate their sensitivity to drugs in vitro, KM12 and drug‐resistant cells were seeded in 96‐well tissue culture plates at 5000 cells/well and incubated at 37°C for 24 h. Cells were incubated with various concentrations of the drugs (0.001, 0.01, 0.1, 1, and 10 μM) or vehicle (dimethyl sulfoxide) at 37°C for 72 h. Cell numbers were estimated using a Cell Counting Kit‐8 (Dojindo, Kumamoto, Japan) and microplate reader (Infinite M200 PRO; Tecan Group Ltd., Männedorf, Switzerland) according to the manufacturer's instructions. The half‐maximal inhibitory concentration (IC_50_) of the drugs tested was defined as the concentration of each agent required for a 50% reduction in cell growth. Each test was conducted independently three times. The corrected absorbance of each sample was calculated and compared with that of the untreated control according to the protocol provided by the manufacturer's instructions.

### 2.3 Antibodies and western blotting

The primary antibodies used were against: pan‐tyrosine kinase inhibitor (TRK; 92991; Cell Signaling Technology, Danvers, MA, USA), phospho‐TRK‐A/B (4619, Tyr490/Tyr516; Cell Signaling Technology), 3‐hydroxy‐3‐methylglutaryl‐CoA synthase 2 (HMGCS2; 137043; Abcam), extracellular signal‐regulated kinase (ERK; 9102; Cell Signaling Technology), phosphor‐ERK (9101, Thr202/Tyr204, Cell Signaling Technology), AKT (9272; Cell Signaling Technology); phospho‐AKT (9271, Ser473; Cell Signaling Technology), 3‐hydroxy‐3‐methylglutaryl‐CoA synthase 2 (HMGCS2; 137043; Abcam), sterol regulatory element‐binding proteins (SREBP2; 13552; Santa Cruz Biotechnology), peroxisome proliferator‐activated receptor alpha (PPARα; ab24509; Abcam, Cambridge, UK), 3‐oxoacid CoA‐transferase 1 (OXCT1; 67836‐1‐lg, Proteintech, Chicago, IL, USA), 3‐hydroxy‐3‐methylglutaryl‐coA lyase (HMGCL; 100548; Santa Cruz Biotechnology), fatty acid synthase (FAS; 48357; Santa Cruz Biotechnology), mammalian target of rapamycin (mTOR; 7C10; Cell Signaling Biotechnology, Dallas, TX, USA), cleaved poly ADP ribose polymerase (cleaved PARP; 5625, Asp214; Cell Signaling Technology), β‐actin (8H10D10; Cell Signaling Biotechnology, Dallas, TX, USA). Protein extraction and western blot analyses were performed as previously described.^19^, ^20^

### 2.4 RNA extraction and quantitative real‐time reverse transcription–PCR

Total RNA was extracted from cultured cells using TRIzol Reagent (Thermo Fisher Scientific, Waltham, MA, USA) as previously described.^21^ The RNA was reverse‐transcribed to cDNA using ReverTra Ace qPCR RT Master Mix (Toyobo, Osaka, Japan) according to the manufacturer's instructions. The quantity and quality of the total RNA were determined using a NanoDrop 2000 spectrophotometer (Thermo Fisher Scientific). The primers, cDNA, and Thunderbird Probe qPCR Mix (Toyobo) were mixed, and quantitative PCR was performed using a 7500 Fast Real‐Time PCR System (Applied Biosystems, San Francisco, CA, USA). *NTRK* (Hs01021011) and HMGCS2 (Hs00985427) gene expression was measured using a TaqMan Gene Expression Assay (Thermo Fisher Scientific) and compared with *GAPDH* (Hs02786624) gene expression as an internal control. The gene expression levels were quantified using the 2−ΔΔCt method.

### 2.5 DNA microarray analysis

Gene expression microarray analysis was performed using a GeneChip Human Gene 2.0 Sense Target array (Affymetrix, Santa Clara, CA, USA), according to the manufacturer's protocol. Microarray data have been deposited in the Gene Expression Omnibus (GEO) at NCBI and are accessible through the GEO series accession number GSE 225681.

### 2.6 Whole exome sequence

DNA was extracted from cultured cell lines using a QIAamp DNA mini kit (QIAGEN, Hilden, Germany) according to the manufacturer's protocol. Exome sequencing of extracted DNA was conducted on an Illumina HiSeq 2500 platform using paired‐end reads (Illumina, San Diego, CA). Reads were aligned against the reference human genome and compared with each cell line. WES data have been deposited in the NCBI Sequence Read Archive under the accession number PRJNA1008352.

### 2.7 Transfection of small interfering RNA

Small‐interfering RNA (siRNA) experiments were performed using Silencer Select Negative Control siRNA as a negative control (4390844; Thermo Fisher Scientific). Pre‐Designed Silencer Select siRNAs were used to knock down HMGCS2 (4392420; Thermo Fisher Scientific). siRNA and Lipofectamine RiMAX Reagent were dissolved in Opti‐MEM Media (Thermo Fisher Scientific) at a final concentration for siRNA complexes of 20 nM. The transfection medium was replaced after 24 h, and the cells were incubated at 37°C for 72 h.

### 2.8 Annexin V assay

Each resistant cells (2.5 × 10^5^ cells/well) with negative control or knocked down HMGCS2 using siRNA were seeded onto six‐well plates. After 24 h, medium was replaced medium containing 1 μM each NTRK‐TKIs incubated at 37°C in 5% CO_2_. After 48 h of incubation, cells were trypsinized, collected, and stained with fluorescein isothiocyanate‐conjugated Annexin V and propidium iodide (PI) using an apoptosis detection kit (Nacalai Tesque Inc.) according to the manufacturer's protocol as previously prescribed.^22^ The cells were analyzed on a BD FACS Verse flow cytometer (10,000 events per sample; BD Bioscience). Fluorescence compensation and analysis were performed with Flow Jo software (BD Bioscience). The percentage of total apoptotic cells, which were both Annexin V‐positive and Annexin V/PI double‐positive cells, was calculated. Each experiment was performed independently three times.

### 2.9 Tumor cell implantation in mice experiments

Female severe combined immunodeficient beige mice (BALB/cAjcl‐nu/nu) were purchased at 5 weeks of age (Charles River Laboratories Japan Inc., Yokohama, Japan). KM12 parental cells and entrectinib‐resistant (KM12‐ER) cells (5 × 10^6^) were injected subcutaneously into the underarms of 6‐week‐old mice. Mice injected KM12 parental cells were randomized to the two cohorts (vehicle control group and entrectinib group) when tumor achieved minimal volume (150–250 mm^3^). The vehicle control group (*n* = 3, per os (p.o.), once a day) and entrectinib group (*n* = 3, p.o., dosed with 15 mg/kg, once a day) were administered until the end of the observation period. Both groups were euthanized after 4 weeks of observation period, and the tumors were removed for investigation. In addition, we performed experiments to confirm the transforming potential and response of NTRK‐TKI‐resistant cells to drugs. In the drug response experiment, mice bearing minimal tumors (150–250 mm^3^) were randomized to the following four cohorts. Vehicle control (*n* = 8, p.o., once a day), simvastatin (*n* = 6, p.o., dosed with 40 mg/kg, once a day), entrectinib (*n* = 6, p.o., dosed with 15 mg/kg, once a day), or both simvastatin, and entrectinib (*n* = 8, p.o., simvastatin dosed with 40 mg/kg, entrectinib dosed with 15 mg/kg, once a day) were administered until the end of the 14 day treatment period. Body weights were measured once per week. Tumor volumes (*V*) were calculated three times a week using caliper measurements of the width (*W*) and length (*L*) of each tumor (*W* ^2^×*L*/2). Statistical analysis was performed based on the measurements on Days 7 and 14 after the drug administration. In conjunction with the IACUC policy, mice were euthanized when the tumor volume was estimated to exceed 10% of body weight, or if the animals showed signs of serious health concerns or suffering.

### 2.10 Immunohistochemical analysis

For immunohistochemical staining of HMGCS2, formalin‐fixed paraffin‐embedded mouse tissue sections were stained by an immunoperoxidase method as previously described.^23^ Slides were incubated with a primary antibody against HMGCS2 obtained from the R and D Systems. Primary antibodies against HMGCS2 (1:100; 137043; Abcam) were used according to the manufacturer's instructions. We also performed Hematoxylin–eosin staining.

### 2.11 Statistical analysis

Differences in categorical outcomes were assessed using the chi‐square test. Statistical significance of differences was determined using a standard Student's *t*‐test. Statistical *p*‐value of <0.05. All analyses were performed using JMP 9 software (SAS Institute, Cary, NC, USA).

## 3 RESULTS

### 3.1 Establishment of NTRK‐TKI–resistant cell lines

**FIGURE 1:**
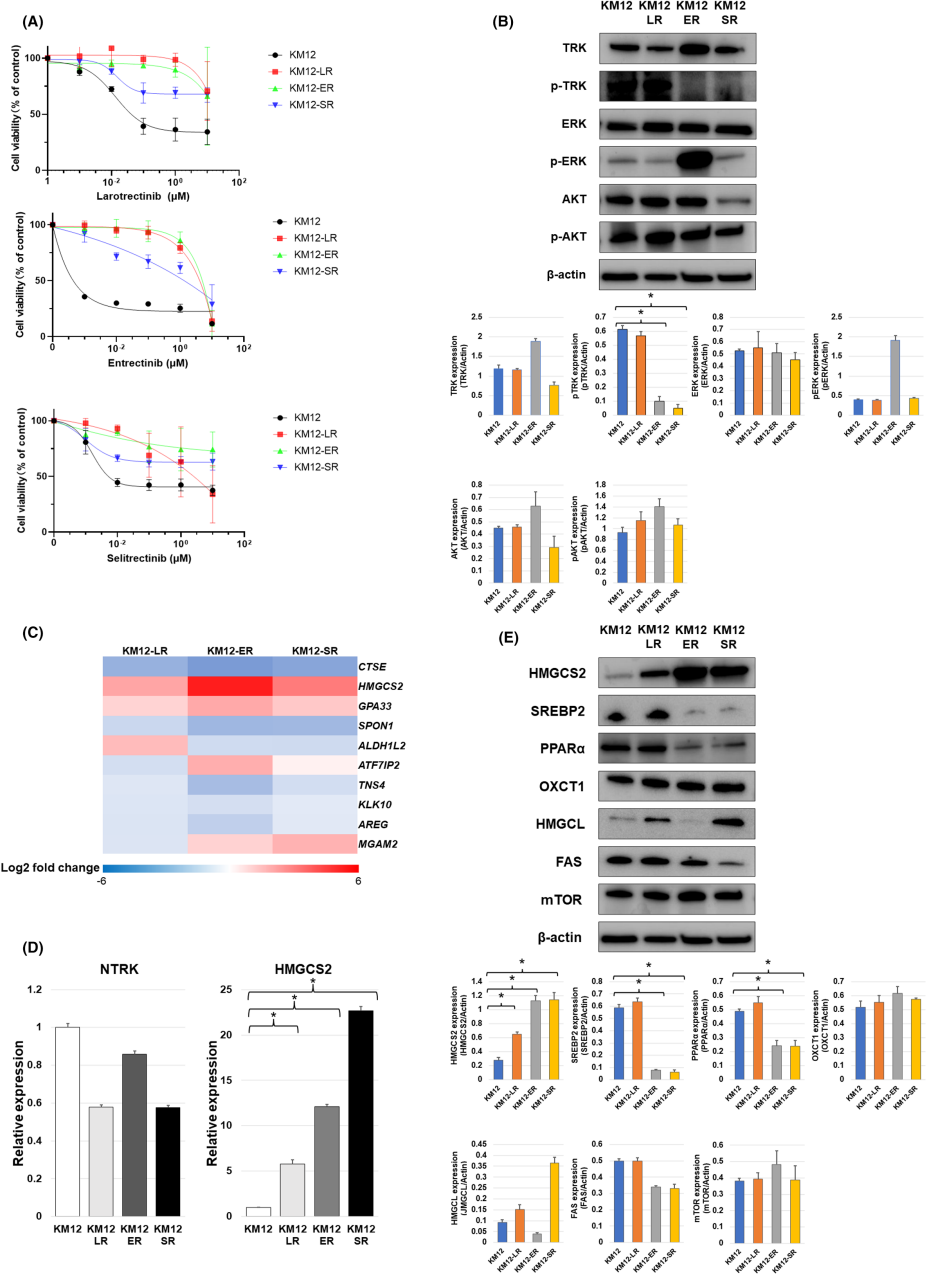
(A) Establishment of cell lines resistant to NTRK‐TKIs (larotrectinib, KM12‐LR; entrectinib, KM12‐ER; selitrectinib, KM12‐SR). The results of the cell viability assay are shown. Each resistant cell line exhibited cross‐resistance to NTRK‐TKIs. (B) Phosphorylation of TRK and downstream signals of the parent and resistant cells were analyzed by western blotting method. Phosphorylation of TRK remained in KM12‐LR, but was lost in KM12‐ER and KM12‐SR. The downstream signals showed activation of ERK phosphorylation in KM12‐ER, but no similar trend was observed in the other resistant cells, and phosphorylation of AKT showed no apparent change in resistant cells compared to parental cell. Protein expression of KM12 parental cell and each resistance cell assessed using Western blotting method was quantified and confirmed, respectively. The band densities were semiquantitatively analyzed using Image J V.1.8.0 (*p < 0.05). (C) Heatmap showed genes with a log2 fold change greater than 1 or less than−1 in common two or more resistant cells in Microarray conducted in NTRK‐TKI–resistant cells compared with parental cells using a log2 ratio [log2 (NTRK‐TKI resistant KM12/parental KM12)] HMGCS2 was commonly upregulated in the three types of resistant cells after assuming the cutoff for log2 fold change was 2.0. (D) The expression of HMGCS2 was increased in NTRK‐TKI‐resistant cells compared with that in parental KM12 cells, as determined by quantitative real‐time reverse transcription–PCR (*p < 0.05). (E) The protein levels of factors related HMGCS2 were analyzed by western blotting. NTRK‐TKI‐resistant cells showed HMGCS2 overexpression. SREBP2 and PPARα related to mevalonate pathway were commonly decreased in KM12‐ER and KM12‐SR which showed stronger HMGCS2 overexpression. Factors related to ketogenesis and fatty acid synthesis showed no common trend or correlation with HMGCS2 expression in the three resistant cells. Protein expression of KM12 parental cell and each resistance cell assessed using Western blotting method was quantified and confirmed, respectively. the band densities were semiquantitatively analyzed using Image J V.1.8.0 (*p < 0.05).

First, we evaluated the sensitivity of the KM12 cells to larotrectinib, entrectinib, and selitrectinib. The IC_50_ values of each drug are presented in Table S1. Based on the IC_50_, we established larotrectinib‐resistant (KM12‐LR), entrectinib‐resistant (KM12‐ER), and selitrectinib‐resistant (KM12‐SR) using stepwise methods over a period of 6 months. We confirmed that all cell lines acquired cross‐resistance to the other TKIs (Table S1; Figure 1A).

### 3.2 HMGCS2 overexpression in NTRK‐TKI‐resistant cell lines

Next, we confirmed NTRK, AKT, and ERK including phosphorylation of these resistant cells. While residual phosphorylation of TRK protein was observed in KM12‐LR cells, loss of TRK phosphorylation was observed in KM12‐ER, and KM12‐SR cells. KM12‐ER showed activation of ERK phosphorylation compared to parental cells (Figures 1B and S1). However, no similar trend was observed in the other resistant cells, and phosphorylation of AKT showed no apparent change in the resistant cells compared to the parental cell (Figures 1B and S1). Microarrays were performed to confirm RNA expression change in resistant cells from parental cells to explore common resistance mechanism independent of NTRK signaling with three resistant cells. Only HMGCS2 was commonly upregulated in the three types of resistant cells after assuming that the cutoff for log2 fold change was 2.0 (Table S2 and Figure 1C). We also performed whole exome sequencing to KM12 parental cells and each resistant cells. A secondary resistance mutation, Gly595Arg, was found in the NTRK1 kinase domain in KM12‐LR. No resistance mutations within the NTRK1,2,3 domains were found in KM12‐ER and KM12‐SR (data are not shown).^10^ Overexpression of HMGCS2 in resistant cells was observed by quantitative RT–PCR and western blotting (Figures 1D,E and S1). In contrast, mRNA expression analysis by RT–PCR showed a trend toward lower expression of NTRK in resistant cells including KM12‐LR with residual phosphorylation of TRK (Figure 1D). Expression of HMGCS2‐related factors, including the mevalonate pathway (PPARα, SREBP2), and fatty acid synthesis (FAS, mTOR) and ketogenesis (OXCT1, HMGCL) were evaluated using western blotting. SREBP2 and PPARα proteins showed no obvious change in KM12‐LR cells compared to parental cells, whereas their expression was clearly decreased in KM12‐ER and KM12‐SR cells. OXCT1, HMGCL, FAS, and mTOR showed no changes or clear trends in levels between parent and resistant cells or correlation with HMGCS2 expression (Figures 1E and S1). Only factors related to the mevalonate pathway (SREBP2 and PPARα) showed a common tendency to correlate with HMGCS2 signal expression. These results suggested HMGCS2 overexpression induced resistance via lipid metabolism, especially the mevalonate pathway, rather than through mechanism through TRK, or ketone metabolism.

### 3.3 Knockdown of HMGCS2 improved the sensitivity to NTRK‐TKIs and mevalonolactone treatment reintroduced resistance to NTRK‐TKIs

**FIGURE 2:**
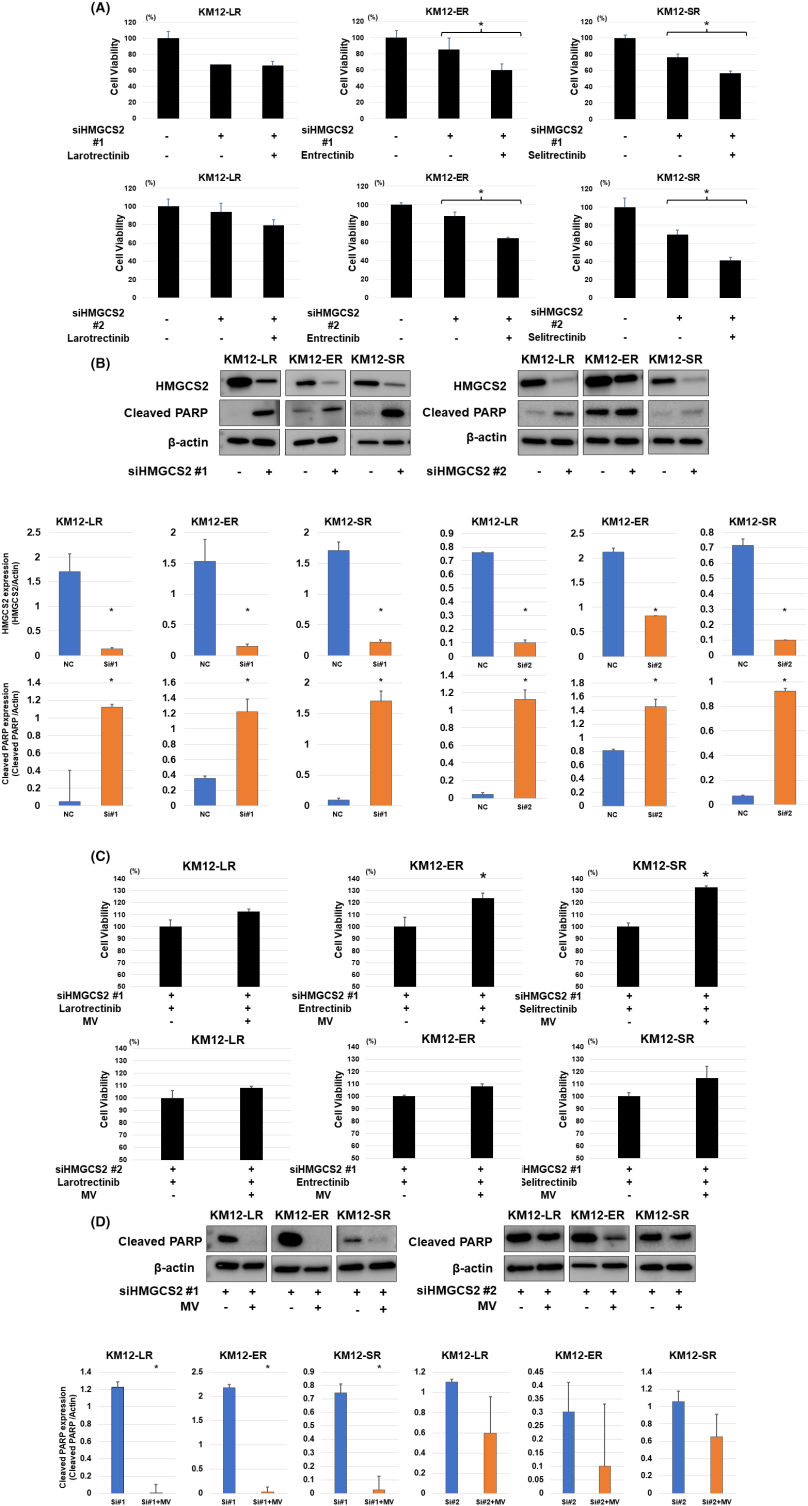
(A) Cell viabilities were significantly reduced in KM12‐ER and KM12‐SR, which had decreased HMGCS2 protein levels in response to HMGCS2 siRNA, after NTRK‐TKI treatment. NTRK‐TKIs were used at 100 nM, respectively, for 72 h (*p < 0.05). (B) After transfection with the two types of siHMGCS2, a decrease in HMGCS2 protein expression and the effect of HMGCS2 knockdown by siRNA to NTRK‐TKI treatment was investigated by evaluating the expression of related protein factors by western blotting. Each NTRK‐TKI treatment was administered at a concentration of 1 μM for 24 h. After HMGCS2 knockdown by siRNA, cleaved PARP levels increased in resistant cells treated with NTRK‐TKIs. (C) An increase in cell viability assay after mevalonolactone and NTRK‐TKI treatment of resistant cells after HMGCS2 knockdown compared to treatment with NTRK‐TKI alone (*p < 0.05). NTRK‐TKIs were used at 100 nM, and mevalonolactone was used at 25 μM for 72 h. (D) After mevalonolactone treatment, cleaved PARP protein levels decreased in resistant cells after HMGCS2 knockdown and NTRK‐TKI treatment. NTRK‐TKIs were used at 100 nM, and mevalonolactone was used at 25 μM for 24 h.

To evaluate the effect of HMGCS2 inhibition on the resistant cells, we suppressed HMGCS2 expression using siRNA. We compared the sensitivity of resistant cells to NTRK‐TKIs after the inhibition of HMGCS2 with siRNA using the cell viability assay. Cell viability was significantly reduced in KM12‐ER and KM12‐SR cells following HMGCS2 siRNA and NTRK‐TKI treatment (Figure 2A). Similar trends in the restoration of NTRK‐TKI sensitivity were observed in KM12‐LR cells, although no significant differences were observed (Figure 2A). HMGCS2 knockdown resulted in decreased cleaved PARP expression following NTRK‐TKI treatment in resistant cells (Figures 2B and S2A). These results suggested knockdown of HMGCS2 induced apoptotic activity by NTRK‐TKI. In comparison, western blotting for downstream signals of TRK and HMGCS2 expression after HMGCS2 knockdown showed no change after siRNA treatment (Figure S3). To demonstrate that HMGCS2 induces resistance via the mevalonate pathway, we assessed the effect of mevalonolactone treatment on NTRK‐TKI‐resistant cells following HMGCS2 knockdown. The cell proliferation assay showed a trend toward the restoration of resistance to the respective NTRK‐TKIs in cells with siRNA knockdown of HMGCS2 after exposure to mevalonolactone (Figure 2C). Furthermore, western blotting for cleaved PARP protein showed that mevalonolactone treatment of resistant cells after HMGCS2 knockdown suppressed apoptotic activity after NTRK‐TKI treatment (Figures 2D and S2B). Annexin V assay confirmed that knockdown of HMGCS2 by siRNA induced apoptosis by NTRK‐TKIs and apoptotic cells was reduced by mevalonolactone (Figure S4).

### 3.4 Overcoming resistance to NTRK‐TKI by simvastatin and silibinin administration

**FIGURE 3:**
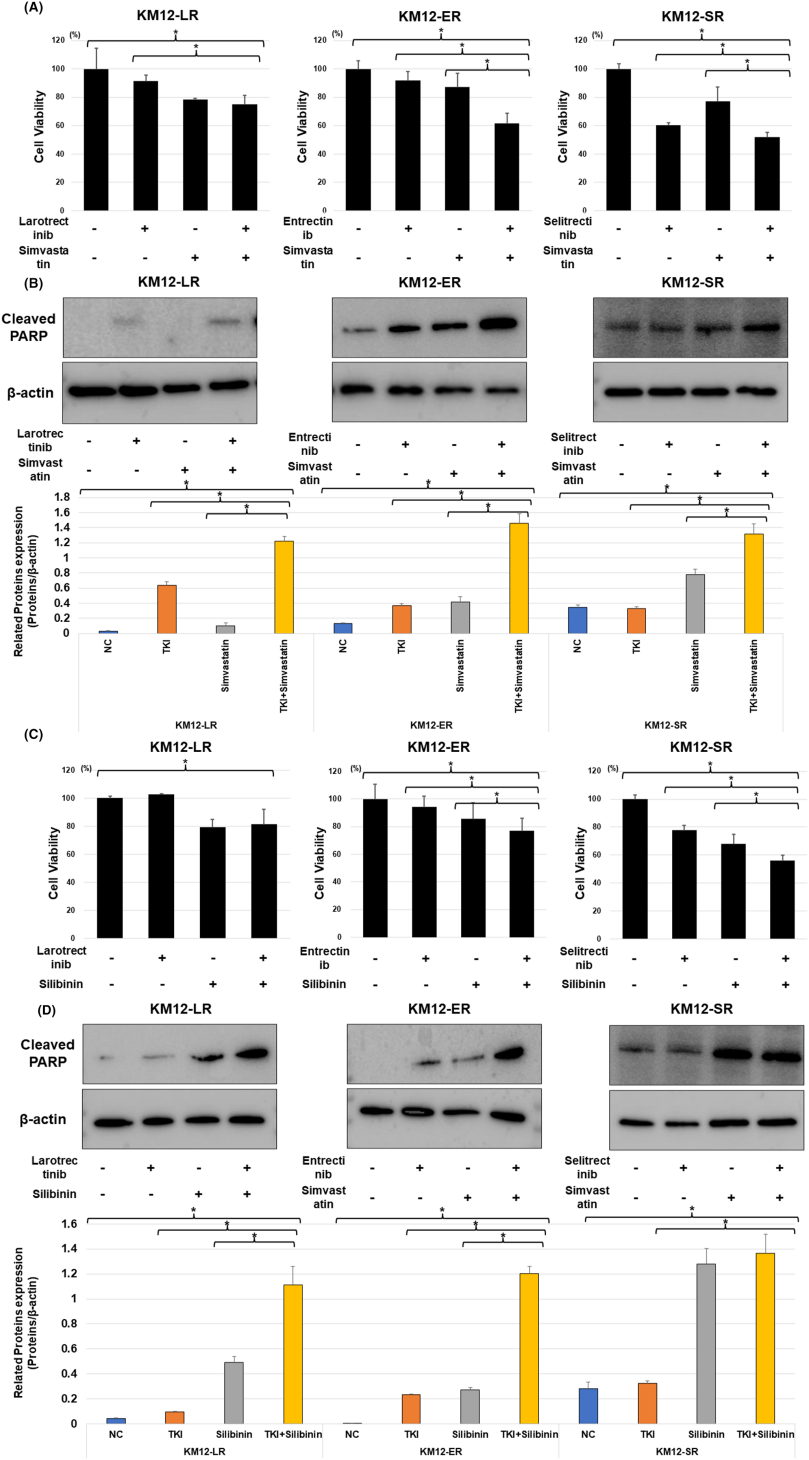
(A) Cell viabilities were significantly reduced in NTRK‐TKI resistant cells after simvastatin and NTRK‐TKIs treatment compared to NTRK‐TKIs alone. Treatments were with 100 nM NTRK‐TKIs (KM12‐LR: larotrectinib, KM12‐ER: entrectinib and KM12‐SR: selitrectinib), 5 μM simvastatin for 72 h (*p < 0.05). (B) Western blotting showed cleaved PARP protein levels were increased in each resistant cell line after treatment with simvastatin and NTRK‐TKIs. Treatments were: 1 μM NTRK‐TKIs (KM12‐LR: larotrectinib, KM12‐ER: entrectinib and KM12‐SR: selitrectinib) and 5 μM simvastatin for 72 h. (C) Cell viabilities were significantly reduced in NTRK‐TKI resistant cells after silibinin and NTRK‐TKIs treatment compared to NTRK‐TKIs alone. Treatments were with 100 nM NTRK‐TKIs (KM12‐LR: larotrectinib, KM12‐ER: entrectinib and KM12‐SR: selitrectinib) and 20 μM silibinin for 72 h (*p < 0.05). (D) Western blotting showed cleaved PARP protein levels were increased in each resistant cell line after treatment with simvastatin and NTRK‐TKIs. Treatments were: (KM12‐LR: larotrectinib, KM12‐ER: entrectinib and KM12‐SR: selitrectinib) 1 μM NTRK‐TKIs and 20 μM silibinin for 72 h. NC, negative control.

We chose simvastatin, an HMG‐CoA reductase inhibitor, and silibinin, known to inhibit HMGCS2 synthesis at the RNA transcription level,^24^ to overcome the induction of HMGCS2 resistance via the mevalonate pathway in previous studies. Treatment of resistant cells with simvastatin was shown to significantly reduce cell viability in resistant cells (Figure 3A). In resistant cells treated with NTRK‐TKI in combination with simvastatin, the levels of cleaved PARP protein were significantly increased compared to those after treatment with each agent alone, reflecting the activation of apoptosis (Figures 3B and S5A). Further experiments were conducted with silibinin to confrm similar results (Figures 3C,D and S5B).

### 3.5 Induction of delayed tolerance to NTRK‐TKI by low concentrations of simvastatin

**FIGURE 4:**
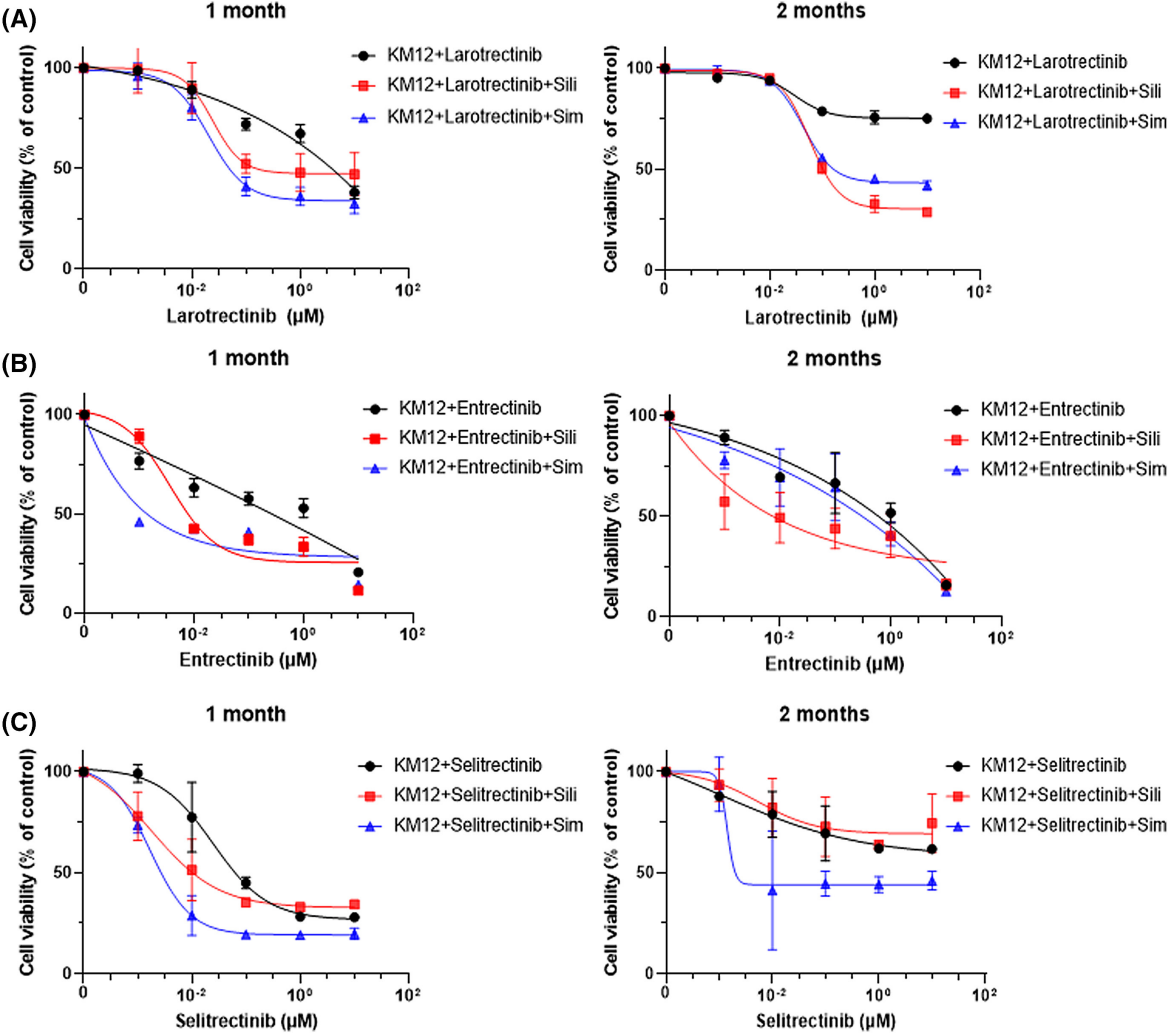
(A–C) Cell viabilities in parental KM12 cells incubated with each NTRK‐TKIs alone and each NTRK‐TKIs plus simvastatin or silibinin for one month and two months were shown. (A: Larotrectinib, B: entrectinib and C: selitrectinib) Cells were continuously incubated with each NTRK‐TKI (50 nM) and simvastatin (25 nM), respectively, which are close to the clinical C max concentration used in human. Silibinin were administered to cells continuously with each NTRK‐TKI at 40 μM. KM12 parental cells treated with combination therapy used each NTRK‐TKIs and simvastatin or silibinin showed a delayed tolerance compared to cells treated with each NTRK‐TKI alone.

We assessed whether a combination of simvastatin or silibinin with NTRK‐TKIs could prevent the acquisition of NTRK‐TKI resistance. At 3 months, resistance was induced by either drug. However, after 1 month, the IC_50_ was significantly lower in cells treated with NTRK‐TKI and simvastatin or silibinin than in those treated with NTRK‐TKI alone (Figure 4A–C and Table S3). After 2 months, similar trends were observed in the relationship between combination treatment and NTRK‐TKI treatment alone (Figure 4A–C).

### 3.6 Simvastatin have potential of reversing entrectinib resistance induced by HMGCS2 via the mevalonate pathway in xenograft mouse models

**FIGURE 5:**
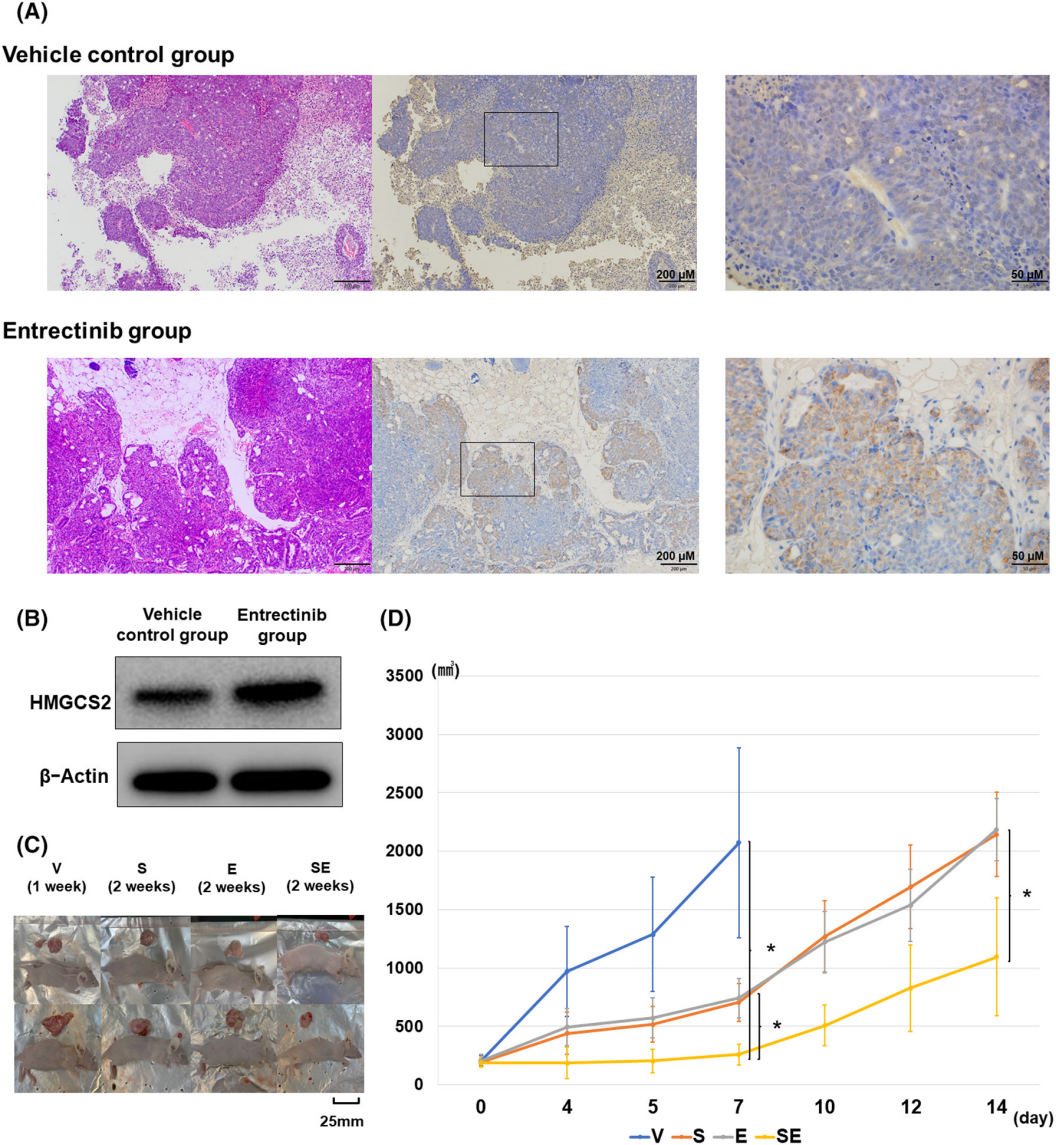
(A) In mouse xenograft models injected with KM12 parental cells, HMGCS2 expression in tumors in vehicle control group (*n* = 3), and those in entrectinib group (*n* = 3) that acquired resistance to entrectinib were compared by immunohistochemistry. Tumors that acquired resistance to entrectinib were shown to HMGCS2 overexpression. (B) Western blot analysis showed HMGCS2 overexpression in KM12 parental cells which acquired resistance to entrectinib in xenograft mouse model compared to the vehicle control. (C) Representatives of mouse xenograft models and the gross appearance of tumors excised at the end of the experiment. Mice of vehicle control group were excised tumor at Day 8, and the other three groups were excised tumor at Day 15. (D) Tumor volumes in xenograft mice transplanted with KM12‐ER cells was significantly lower in the simvastatin and entrectinib combination and entrectinib treatment groups when comparing tumor volumes on Days 7 and 14 (Vehicle control group *n* = 8, Simvastatin group *n* = 6, Entrectinib group *n* = 6, Simvastatin, and entrectinib group *n* = 8). At Day 7, 14 after started treatment, combination treatment with entrectinib and simvastatin significantly reduced tumor volume compared to treatment with entrectinib alone (*p < 0.05). E, Entrectinib group; S, Simvastatin group; SE Simvastatin and entrectinib group; V, Vehicle control group.

Of xenograft mouse models with KM12 parental cells, the entrectinib group showed tumor volume decrease after 1 week started treatment. After 28 days started entrectinib treatment, 1 of 3 mice of the entrectinib group showed tumor resistance (Figure S6A). Tumor that became resistant to entrectinib in mouse showed overexpression of HMGCS2 compared to the vehicle control, which was confirmed by immunohistochemistry (IHC) and western blot analysis (Figure 5A,B). We used xenograft mouse models with KM12‐ER cells to confirm the effect of NTRK‐TKI and simvastatin. Tumor volumes of xenograft mice injected with KM12‐ER cells were significantly lower after treatment with simvastatin in combination with vehicle group and entrectinib group, after comparing tumor volumes at day 7 and day 14 (Figures 5C,D and S6B). In vivo, simvastatin and NTRK‐TKI showed a synergistic effect on cells that acquired resistance to NTRK‐TKI via HMGCS2.

## 4 DISCUSSION

HMGCS2 is a key enzyme in ketogenesis, and several previous studies have suggested an inhibitory effect on tumor growth, migration, and angiogenesis through ketone synthesis in mitochondria.^25^, ^26^, ^27^, ^28^, ^29^, ^30^, ^31^ Low HMGCS2 expression is a poor prognostic factor in colorectal cancer, esophageal squamous cell carcinoma, prostate cancer, and hepatocellular carcinoma.^28^, ^29^, ^32^, ^33^ Ketone production by HMGCS2 is generally considered to be an inhibitory regulatory mechanism in tumors. In contrast, previous research showed that HMGCS2 enhances invasion and metastasis by directly interacting with PPARα to activate Src signaling in colorectal and oral cancers. Additionally, HMGCS2 overexpression is a poor prognostic factor, along with other lipid metabolism–related factors.^30^ In prostate cancer, HMGCS2 and sterol synthesis have been implicated in tumor growth and progression by co‐culture with cancer‐associated fibroblasts.^34^ HMGCS2 is associated with cholesterol synthesis via the mevalonate pathway by producing HMG‐CoA in peroxisomes other than during ketogenesis.^35^, ^36^ These results suggested that HMGCS2 may contribute to cancer progression through lipid metabolism, including the mevalonate pathway.

Several mechanisms have been reported for the resistance to anticancer drugs via the mevalonate pathway. The mevalonate pathway activates isoprenylation of geranylgeranyl pyrophosphate (GGPP), which induces activation of the small GTP‐binding protein family and the YAP1/TAZ pathway, and acts as an off‐target resistance mechanism against TKIs.^37^, ^38^, ^39^ Cholesterol, the end‐product of the mevalonate pathway, is known to contribute to cancer cell proliferation by altering cellular functions through the regulation of many signaling pathways and the metabolic byproduct oxysterol.^40^ As HMG‐CoA reductases to suppress these pathways, thereby inhibiting cancer growth and drug resistance.^37^, ^41^, ^42^ Previous in vitro studies have reported that pitavastatin has synergistic efficacy with erlotinib and mevalonic acid treatment by introducing resistance in de novo EGFR‐TKI‐resistant cell lines.^43^, ^44^ Recently, in a non‐small cell lung cancer harboring an *EGFR* mutation, a large retrospective cohort study showed that progression‐free survival was longer in patients who received statins to treat dyslipidemia, for example, than in patients who received EGFR‐TKI alone,^45^ suggesting that the relationship between molecular targeted therapies and the control of lipid metabolism is of interest. The relationship between molecular targeted therapies and the control of lipid metabolism has been the focus of much attention.

This study is the first to suggest that HMGCS2 overexpression is related to resistance to anticancer agents via the mevalonate pathway. Furthermore, in xenograft mouse models, simvastatin and entrectinib were shown to have synergistic antitumor effects against entrectinib‐resistant tumors. In addition, the inhibition of HMGCS2, not only by simvastatin, but also by silibinin, a drug with a mechanism different from that of statins, produced results similar to those of statins, reinforcing the present results. Our study showed that simvastatin is a promising treatment for HMGCS2‐induced resistance and may affect tolerance delay when used in combination with NTRK‐TKI at clinical concentrations in an NTRK‐rearranged tumor. Numerous studies have described the anticancer effects of statins, but most of the data have been reported at much higher doses of statins compared to doses for humans. However, our results presented that statins at the concentrations used in clinical practice may be effective in delaying the development of drug resistance, although further studies are required.

**FIGURE 6:**
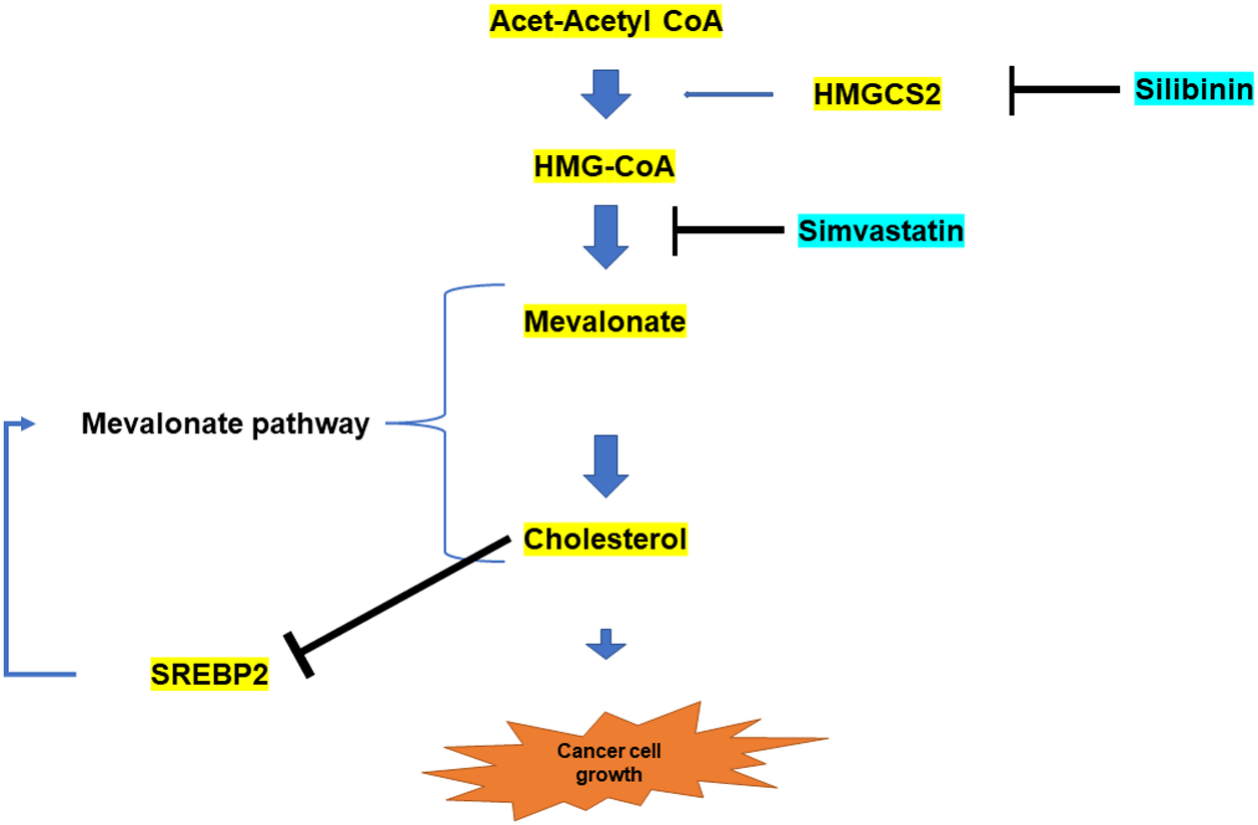
Schematic result in this study. HMGCS2 induces resistance to NTRK‐TKI via the mevalonate pathway, suggesting that simvastatin may overcome this resistance.

Interestingly, even in KM12‐LR, in which a resistant mutation was confirmed by whole exome sequencing, HMGCS2 expression was upregulated compared to the parental cells. Furthermore, in KM12‐LR, phosphorylation of TRK was not affected by HMGCS2 knockdown. The results of the cell viability assay suggested that HMGCS2 knockdown and synergistic effects with TKI by statin and silibinin administration tended not to be obtained as in the other two resistant cells. This may be due to the presence of a secondary resistance mutation in KM12‐LR that affects the synergistic effect. These indicate that HMGCS2 overexpression is responsible for resistance to NTRK‐TKI independently from secondary resistance mutations. In addition, these results suggested our study showed not an additive effect resulting from the anti‐tumor effect of statins, but resulting from statin induce overcoming resistance mechanism of NTRK‐TKIs from HMGCS2 in our study. As mentioned above, there are data suggesting that the mechanism of resistance by activation of the mevalonic acid pathway may also be possible with other TKIs, and it will be necessary to confirm in the future whether this mechanism works not only with NTRK‐TKIs but also with other TKIs. The results of our study are presented in a schematic (Figure 6).

Our study has some important limitations. Unfortunately, due to its rarity, no clinical specimens or clinical cases of NTRK fusion‐positive tumors could be identified in our laboratory. HMGCS2 overexpression associated with a drug‐tolerant subpopulation was observed in single cell line harboring an NTRK rearrangement. In addition, although it has been shown that resistance is induced through the mevalonate pathway, the details of the mechanism could not be elucidated. Confirmation of the details of the resistance mechanism via the mevalonate pathway could have a further impact on overcoming further resistance mechanisms with any kinds of TKI and needs to be validated in future. Our study highlights that overexpression or knockdown of HMGCS2 does not affect AKT and ERK expression and phosphorylation. These results suggest that a different mechanism is at work than the activation of G protein families which is generally considered to be involved in drug resistance and cancer progression through the mevalonate pathway. We plan to conduct further research to address these issues.

## 5 CONCLUSION

This study is a first report showed that HMGCS2 promotes the induction of resistance, common to first‐ and second‐generation NTRK‐TKIs, through the mevalonate pathway. In addition, inhibiting HMGCS2 or the mevalonate pathway may show promise in overcoming HMGCS2‐induced resistance to NTRK‐TKIs or delaying the development of drug resistance. Therefore, statins may be a promising therapeutic strategy in patients with NTRK rearrangements in both initial and recurrent treatment phases. Further studies should be performed on the significance of HMGCS2 and statins in a drug‐tolerant cancer cell subpopulation.

## AUTHOR CONTRIBUTIONS

**Masaru Matsumoto:** Conceptualization (lead); data curation (lead); investigation (lead); methodology (lead); writing – original draft (equal); writing – review and editing (equal). **Yasuhiro Kato:** Conceptualization (lead); data curation (lead); formal analysis (lead); investigation (lead); methodology (lead); project administration (lead); validation (lead); writing – original draft (lead). **Natsuki Takano:** Conceptualization (supporting); data curation (equal); investigation (supporting); methodology (equal); writing – review and editing (supporting). **Mariko Hirao:** Investigation (equal); methodology (equal); resources (equal); validation (equal); writing – review and editing (equal). **Kuniko Matsuda:** Data curation (equal); investigation (equal); methodology (equal); resources (equal); validation (equal). **Takehiro Tozuka:** Supervision (equal); writing – review and editing (equal). **Naomi Onda:** Supervision (equal); writing – review and editing (equal). **Shinji Nakamichi:** Methodology (supporting); supervision (equal); writing – review and editing (equal). **Susumu Takeuchi:** Supervision (equal); writing – review and editing (equal). **Akihiko Miyanaga:** Data curation (equal); investigation (equal); methodology (equal); project administration (equal); supervision (lead); writing – original draft (equal); writing – review and editing (equal). **Rintaro Noro:** Data curation (equal); formal analysis (equal); methodology (equal); supervision (equal); writing – review and editing (equal). **Akihiko Gemma:** Conceptualization (equal); funding acquisition (lead); supervision (equal); writing – review and editing (equal). **Masahiro Seike:** Conceptualization (lead); funding acquisition (lead); project administration (lead); resources (lead); supervision (lead); writing – review and editing (lead).

## FUNDING INFORMATION

This study was supported in part by the Clinical Rebiopsy Bank Project for Comprehensive Cancer Therapy Development from the Ministry of Education, Culture, Sports, Science, and Technology Supported Program for the Strategic Research Foundation at Private Universities (grant 16K09592 to M. Seike and grant S1311022 to A. Gemma).

## CONFLICT OF INTEREST STATEMENT

MS received honoraria for lectures and research funding from Chugai Pharmaceutical. AG received honoraria for manuscript‐fee funding from Chugai Pharmaceutical. SN and TT received honoraria for their lecture fees from Chugai Pharmaceutical. The authors declare no conflicts of interest.

## ETHICS STATEMENT

Approval of the research protocol by an Institutional Reviewer Board: N/A.

## CONSENT

*Registry* *and the Registration no*. *of the study*: N/A.

*Animal studies*: Animal studies of this study were approved by the Institutional Animal Care and Use Committee (Permission number: 2022‐057) and carried out according to the Nippon Medical School Animal Experimentation Regulation.

## Supporting information


Figure S1.
Figure S2.
Figure S3.
Table S1.
Table S2.
Table S3.

## Data Availability

I confirm that my article contains a Data Availability Statement even if no data is available (list of sample statements) unless my article type does not require one. I confirm that I have included a citation for available data in my references section, unless my article type is exempt.
