# Correction to “Induction of Resistance to Neurotrophic Tropomyosin‐Receptor Kinase Inhibitors by HMGCS2 via a Mevalonate Pathway”

**DOI:** 10.1002/cam4.70350

**Published:** 2024-11-28

Yasuhiro K, Masaru M, Natsuki T, Mariko H, Kuniko M, Takehiro T, Naomi O, Shinji N, Susumu T, Akihiko M, Rintaro N, Akihiko G, Masahiro S, “Induction of Resistance to Neurotrophic Tropomyosin‐Receptor Kinase Inhibitors by HMGCS2 via a Mevalonate Pathway” Cancer Medicine, Volume 13, no. 12 (2024): e7393. https://doi.org/10.1002/cam4.7393.

We have replaced the β‐actin of KM12‐SR after knockdown HMGCS2 using siHMGCS2#2 in Figure 2D with the correct Figure.

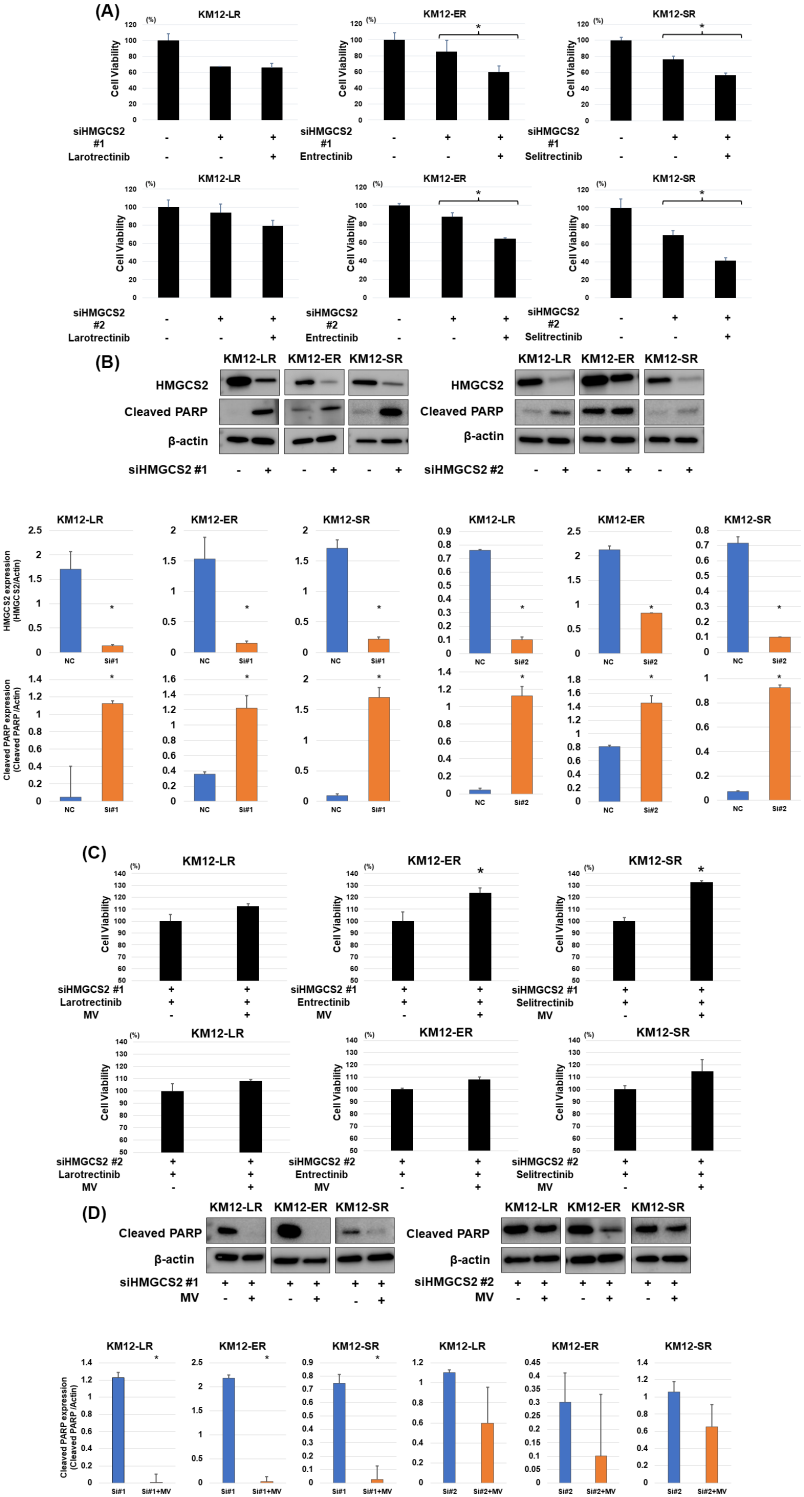

We apologize for this error.

