# Supplementary material for: Induction of resistance to neurotrophic tropomyosin‐receptor kinase inhibitors by HMGCS2 via a mevalonate pathway
Source: Cancer Med. 2024 Jun 24;13(12):e7393. doi: 10.1002/cam4.7393 (PMC11194613; doi:10.1002/cam4.7393)
Supplement: Supplementary file 1 — Figure S1. Figure S2. Figure S3. Table S1. Table S2. Table S3. [file CAM4-13-e7393-s001.docx]

The below given format is the standard format of presenting supplementary information in Cancer Science and we apologise for any inconvenience caused by this procedure.

This format can be presented in either doc / xlx / pdf/ tif/ JEG / EPS, ensure the given supplementary file is without any highlights or track changes and the given supporting information is cited in the main document.

Supplemental tables

Table S1 IC50 of parental and NTRK-TKI resistant cells in KM12 cell line

| IC50 | Larotrectinib | Entrectinib | Selitrectinib | (μM) |
| --- | --- | --- | --- | --- |
| KM12 | 0.046 | 0.0008 | 0.0086 |  |
| KM12-ER | >10 | 5.02 | 5.04 |  |
| KM12-LR | >10 | 8.89 | >10 |  |
| KM12-SR | >10 | 4.15 | >10 |  |

All experiments were performed three times using MTT assays.

Abbreviations: ER, entrectinib resistance; IC50, half maximal inhibitory concentration; LR, larotrectinib resistance; MTT, tetrazolium; NTRK-TKI, neutrophic tropomyosin-receptor kinase–tyrosine-kinase inhibitor; SR, selitrectinib resistance

Table S2 The result of microarray analysis showed HMGCS2 overexpression in NTRK-TKI resistant cells

|  | KM12 | KM12-LR | KM12-ER | KM12-SR |  |
| --- | --- | --- | --- | --- | --- |
| CTSE | 1 | -3.32 | -4.22 | -3.79 | (Log2 fold change) |
| HMGCS2 | 1 | 2.14 | 5.34 | 3.12 |  |
| GPA33 | 1 | 1.04 | 2.04 | 1.30 |  |
| SPON1 | 1 | -1.67 | -3.05 | -3.00 |  |
| ALDH1L2 | 1 | 1.62 | -1.52 | -1.55 |  |
| ATF7IP2 | 1 | -1.40 | 1.92 | 0.34 |  |
| TNS4 | 1 | -1.06 | 2.81 | -1.42 |  |
| KLK10 | 1 | -1.10 | 1.37 | -0.91 |  |
| AREG | 1 | -1.13 | -2.02 | -0.96 |  |
| MGAM2 | 1 | -1.12 | 1.07 | 1.82 |  |

ER, entrectinib resistance; HMGCS2, 3-hydroxy-3-methylglutaryl-CoA synthase 2; LR, larotrectinib resistance; NTRK-TKI, neutrophic tropomyosin-receptor kinase–tyrosine-kinase inhibitor; SR, selitrectinib resistance

Table S3 IC_50_ after 1, 2, or 3 months of continuous exposure of KM12 cells to NTRK-TKIs alone, NTRK-TKIs + silibinin, or simvastatin

|  | 1 month | P-value | 2 months | P-value | IC50(µM) |
| --- | --- | --- | --- | --- | --- |
| Larotrectinib (50 nM) | 6.3±0.64 |  | >10 |  |  |
| Larotrectinib (50 nM) +Silibinin (40 µM) | 0.079±0.0013 | <0.001 | 0.59±0.089 | 0.01 |  |
| Larotrectinib (50 nM) +Simvastatin (25 nM) | 0.24±0.054 | 0.001 | 0.13±0.062 | 0.01 |  |
|  |  |  |  |  |  |
| Entrectinib (50 nM) | 0.98±0.59 |  | 1.79±0.65 |  |  |
| Entrectinib (50 nM) +Silibinin (40 µM) | 0.00093±0.0005 | 0.049 | 0.05±0.036 | 0.02 |  |
| Entrectinib (50 nM) +Simvastatin (25 nM) | 0.0086±0.0007 | 0.049 | 0.55±0.049 | 0.054 |  |
|  |  |  |  |  |  |
| Selitrectinib (50 nM) | 0.085±0.000076 |  | >10 |  |  |
| Selitrectinib (50 nM) +Silibinin (40 µM) | 0.0066±0.00026 | ＜0.001 | >10 | 0.48 |  |
| Selitrectinib (50 nM) +Simvastatin (25 nM) | 0.00762±0.0018 | ＜0.001 | 0.075±0.0030 | <0.001 |  |

All experiments were performed three times using MTT assays.

Abbreviations: IC_50_, inhibitory concentration showing a 50% response; MTT, tetrazolium; NTRK-TKI, neutrophic tropomyosin-receptor kinase–tyrosine-kinase inhibitor;

Supplimentaly figure

Figure S1


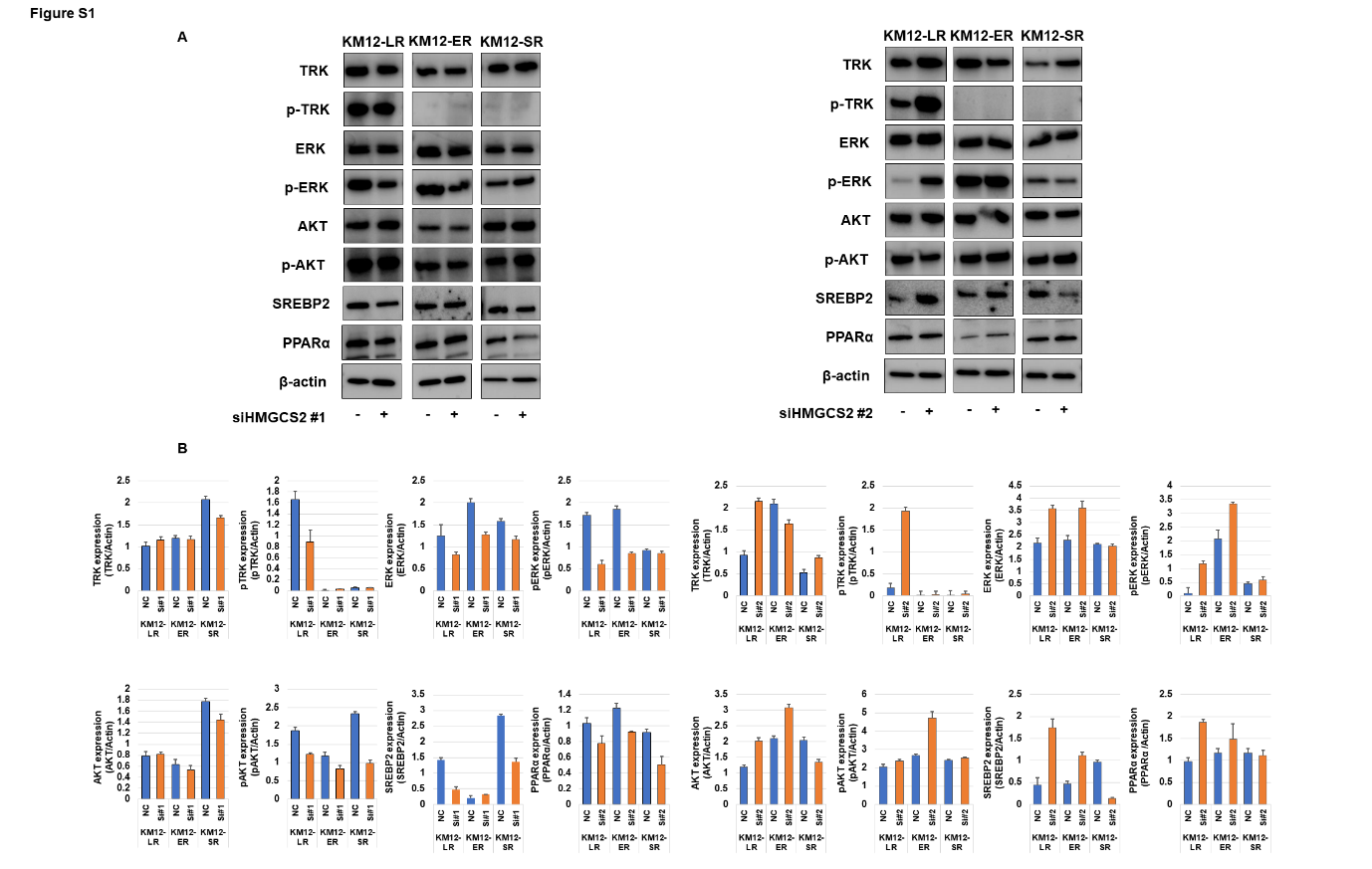


A, B) We investigated protein expression change related TRK and mevalonate pathway after knockdown of HMGCS2. No consistent trend was observed for any of the factors.

Figure S2
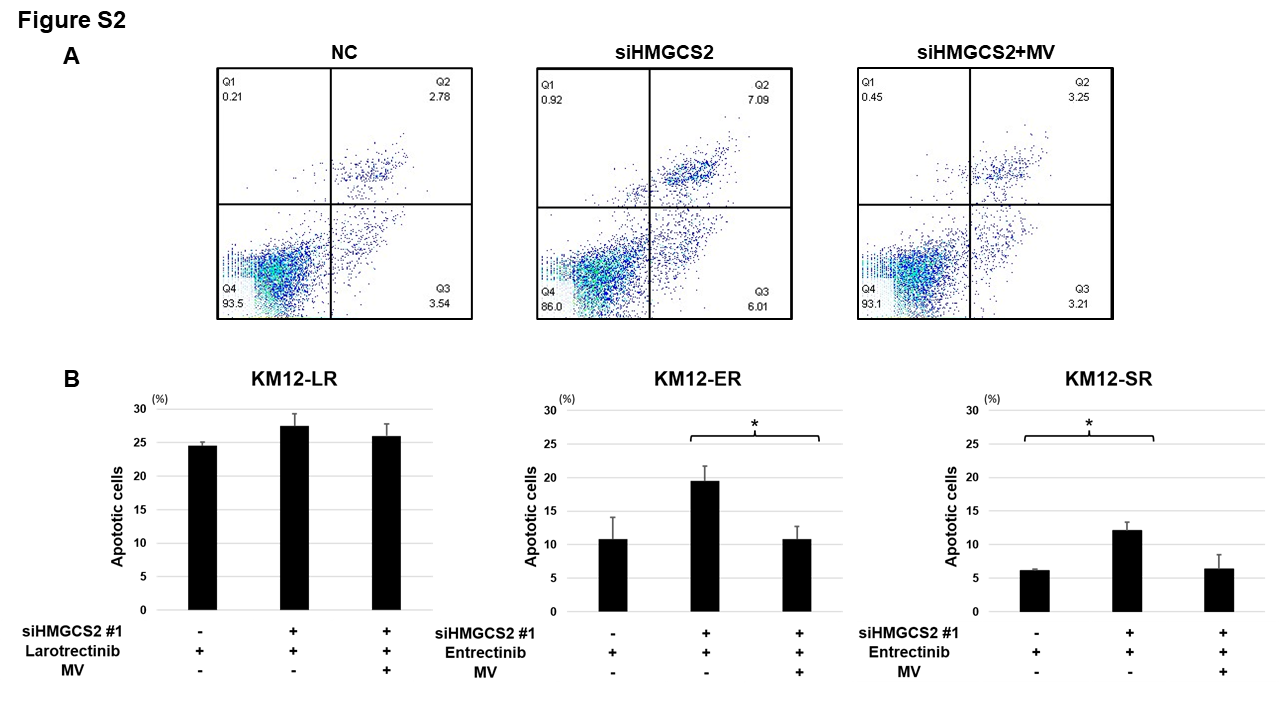


1. Representative annexin V assays using KM12-ER are shown.
2. The apoptotic activity in resistant cells were confirmed using Annexin V assay. The percentage of apoptotic cells was significantly increased by siHMGCS2 over normal control and significantly decreased with the addition of 25µM mevalonolactone. All cells were treated with 1 µM NTRK-TKI. (**p* < 0.05).

Abbreviation: MV, mevalonolactone

Figure S3
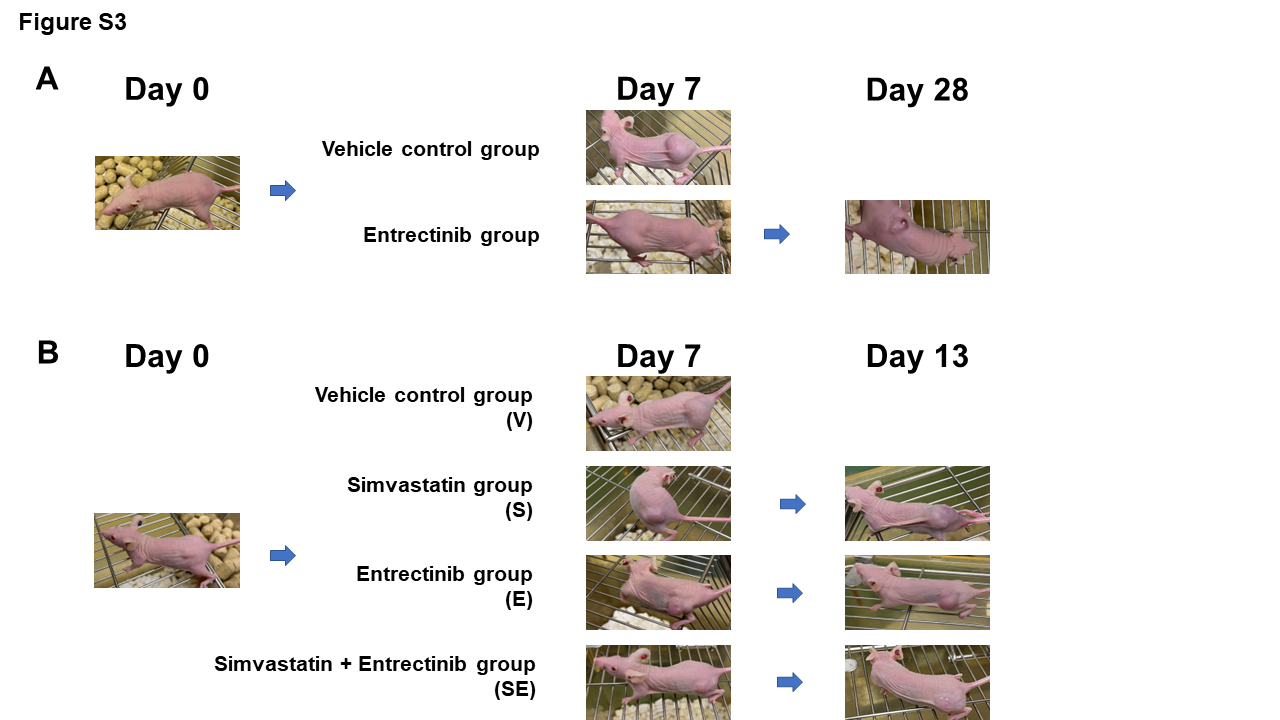


1. Progress of mice transplanted with KM12 parental cells. Tumours increased in vehicle control group and required euthanasia within a week; in entrectinib group, treatment continued to suppress tumor growth, but an enlarged tumor was observed after 4 weeks.
2. Representative progression of each group in the xenograft mouse model transplanted KM12-ER. The combination treatment group was observed to suppress tumor growth more than entrectnib and simvastatin groups.
